# MRI-related anxiety in healthy individuals, intrinsic BOLD oscillations at 0.1 Hz in precentral gyrus and insula, and heart rate variability in low frequency bands

**DOI:** 10.1371/journal.pone.0206675

**Published:** 2018-11-26

**Authors:** Gert Pfurtscheller, Andreas Schwerdtfeger, David Fink, Clemens Brunner, Christoph Stefan Aigner, Joana Brito, Alexandre Andrade

**Affiliations:** 1 Institute of Neural Engineering, Graz University of Technology, Graz, Austria; 2 BioTechMed, Graz, Austria; 3 Department of Psychology, University of Graz, Graz, Austria; 4 Institute of Medical Engineering, Graz University of Technology, Graz, Austria; 5 Institute of Biophysics and Biomedical Engineering, Faculty of Sciences of the University of Lisbon, Lisbon, Portugal; McMaster University, CANADA

## Abstract

Participation in magnetic resonance imaging (MRI) scanning is associated with increased anxiety, thus possibly impacting baseline recording for functional MRI studies. The goal of the paper is to elucidate the significant hemispheric asymmetry between blood-oxygenation-level-dependent (BOLD) signals from precentral gyrus (PCG) and insula in 23 healthy individuals without any former MRI experience recently published in a PLOSONE paper. In addition to BOLD signals state anxiety and heart rate variability (HRV) were analyzed in two resting state sessions (R1, R2). Phase-locking and time delays from BOLD signals were computed in the frequency band 0.07–0.13 Hz. Positive (pTD) and negative time delays (nTD) were found. The pTD characterize descending neural BOLD oscillations spreading from PCG to insula and nTD characterize ascending vascular BOLD oscillations related to blood flow in the middle cerebral artery. HRV power in two low frequency bands 0.06–0.1 Hz and 0.1–0.14 Hz was computed. Based on the anxiety change from R1 to R2, two groups were separated: one with a strong anxiety decline (large change group) and one with a moderate decline or even anxiety increase (small change group). A significant correlation was found only between the left-hemispheric time delay (pTD, nTD) and anxiety change, with a dominance of nTD in the large change group. The analysis of within-scanner HRV revealed a pronounced increase of low frequency power between both resting states, dominant in the band 0.06–0.1 Hz in the large change group and in the band 0.1–0.14 Hz in the small change group. These results suggest different mechanisms related to anxiety processing in healthy individuals. One mechanism (large anxiety change) could embrace an increase of blood circulation in the territory of the left middle cerebral artery (vascular BOLD) and another (small anxiety change) translates to rhythmic central commands (neural BOLD) in the frequency band 0.1–0.14 Hz.

## Introduction

A major challenge associated with magnetic resonance imaging (MRI) scans performed with closed bore systems is the placement in a narrow place producing discomfort and even extensive fear. It is estimated that 25–37% of patients experience moderate anxiety [1] and about 2% intensive anxiety [2] during clinical scans. Although MRI-related anxiety and therewith activation of specific brain structures is common, little is known about their impact on slow intrinsic frequency fluctuations and resting state functional connectivity, respectively, in the frequency range near 0.1 Hz. A study on MRI-naïve young men with self-reported anxiety revealed highest anxiety levels during the first scan with a drop in the second scan [3]. MRI-related anxiety can affect the connectivity between the default mode network and left insula in youth and adults [4] and should affect neural and vascular blood-oxygenation-level-dependent (BOLD) oscillations with dominant frequencies near 0.1 Hz in different ways.

The discrimination between neural and vascular BOLD oscillations at approximately 0.1 Hz is possible through computing the phase-locking value (PLV), either between two BOLD signals [5] or between BOLD and heart rate beat-to-beat interval (RRI) signals [6,7]. By measuring phase-shifts of slow BOLD oscillations between two regions of interest (ROIs) and determining the sign of time delay (positive or negative) it is feasible to discriminate between ascending slow BOLD oscillations driven by the Mayer waves in cerebral blood flow and blood pressure (vascular BOLD) and descending BOLD oscillations most likely associated with neural activity fluctuations (neural BOLD; [5]).

Although anxiety processing is strongly related to the activation of amygdala (AMY) and related regions ([8], [9], [10]), the present study investigates BOLD signals from precentral gyrus (PCG) and insula (INS) since these two ROIs were successfully used to discriminate between neural and vascular BOLD oscillations near 0.1 Hz [5]. The INS is a brain structure implicated in many regulatory functions including emotional responses and cardiac control ([4], [11]) and connected bidirectionally with the PCG an area important for planning and execution of motor behavior [12]. Both PCG and INS are supplied by branches of middle cerebral artery (MCA) a prerequisite to study cerebral circulation with BOLD signals, as cerebral blood flow velocity (CBFv) is largest in MCA compared with the other main cerebral arteries [13]. Although the AMY is one of the key regions of the limbic system and related to anxiety processing, it is not optimal suited for PLV measurements because of its poor signal quality through magnetic inhomogeneities and the comparably small number of voxels when the AAL atlas is used [14].

There is an enduring interest in brain oscillations at 0.1 Hz and 10-s waves in blood pressure, heart rate and respiration, respectively, documented by two important papers published recently. One reports on entrainment of arteriole vasomotor and BOLD oscillations by gamma band activity [15] and the other on resonance breathing at a 10-s rate and “emotional well-being” [16]. The former contributes to the genesis of slow intrinsic neural BOLD oscillations near 0.1 Hz and the latter helps to explain the importance of low frequency HRV in emotion regulation and especially anxiety processing [17, 18].

In a previous PLOSEONE paper about the distinction between neural and vascular BOLD oscillations at 0.1 Hz [5] we reported on a significant hemispheric asymmetry during rest in healthy, scanner-naïve subjects, without providing a satisfactory explanation about their origin. The goal of the present paper is the attempt to explain this hemispheric asymmetry by considering not only the elevated state anxiety (AS) and its change between two consecutive resting states (anxiety change) but also the variation in time of the low frequency HRV in two bands.

## Methods

### Participants

From a total of 25 individuals (12 female) between 19–34 years (mean ± SD: 24 ± 3.2 years) two were excluded due to cardiac arrhythmia. All were naïve to the purpose of the study, had no former MRI experience, had normal or corrected-to-normal vision and were without any record of neurological or psychiatric disorders as assessed via self-report. All individuals gave informed written consent to the study protocol, which had been approved by the local Ethics Committee at the University of Graz.

### Experimental protocol

The experimental task consisted of two resting states (R1, R2) and two within-scanner questionnaires on state anxiety (AS1, AS2) separated by about 30 minutes. The task started with the first questionnaire (AS1) and was followed by the first resting state (R1). Thereafter, two movement tasks (each lasting 600 s) were performed. The session ended with the second resting state (R2) and second questionnaire (AS2). Filling out each questionnaire took approximately 5 minutes and each resting state lasted for about 350 s. Individuals were requested to keep their eyes open, to stay awake, and to avoid movements during the resting states.

State anxiety was assessed with the state version of the state-trait anxiety and depression inventory (STADI; [19], which was presented on a screen within the scanner. The STADI is an instrument constructed to assess both state and trait aspects of anxiety and depression. It is based on the State-Trait Anxiety Inventory [20], but allows a reasonable separation of anxiety and depression symptoms. Items were answered with a trackball following each resting state.

### fMRI data acquisition and preprocessing

Functional images were acquired on a 3 T scanner (Magneton Skyra, Siemens). A multiband GE- EPI sequence [21] was applied with the following parameters: multiband factor 6, voxel size 2x2x2 mm³, TR/TE = 871/34 ms, flip angle 52 degrees, matrix 90x104, 66 contiguous axial slices, FOV = 180x208 mm^2^. Pre-processing and region of interest (ROI) signal extraction was performed using the DPARSF toolbox [22]. Pre-processing included the removal of the first 10 volumes, slice- timing correction adapted for multiband acquisitions, rigid-body motion correction, normalization to Montreal Neurological Institute (MNI) space, resampling to 2-mm isotropic voxels, spatial smoothing with a 4-mm FWHM Gaussian kernel and linear detrending. Lastly, the BOLD time courses of left and right precentral gyrus (PCG) and left and right insula were extracted, as defined in the Automated Anatomical Labeling (AAL) atlas [23]. For further details see Pfurtscheller et al. [5].

### HRV analysis

ECG and respiration were recorded inside the scanner with a sampling rate of 400 Hz. After heart beat detection using fMRI plug-in for EEGLAB [24] and calculation of beat-to-beat interval (RRI) time course (sample rate 4 Hz) the time course was saved as text file and imported to the KUBIOS HRV Premium Package (Kubios Ltd. Finland; version 3.0.2) [25]. Spectral analyses using a Fast Fourier transform algorithm with a window width of 125 s window with 50% overlap was applied to determine the frequency domain estimates of low frequency (LF) HRV in the two bands 0.06–0.1 Hz (LFa) and 0.1–0.14 Hz (LFb). For statistical analysis, natural log transformed power (log power) and relative power (% power) were used.

### BOLD data processing

For processing of BOLD signals the “Cross-wavelet and Wavelet Coherence” toolbox [26] was used. After bandpass filtering between 0.07 and 0.13 Hz the phase-locking value (PLV) was computed for the two pairs of BOLD signals from PCG and insula and the parameters time delay (TD) and significant length of phase coupling (*%sigbins*) were extracted. A positive time delay (pTD) indicates a lead and a negative delay (nTD) a lag of BOLD oscillations at 0.1 Hz in PCG compared to oscillations in insula. The percentage of significant (p<0.05) time samples or bins (%sigbins), which indicates the total length of significant phase-locking episode, was also computed in each case. For further details see Pfurtscheller et al. [5].

### Interlinkage between different analyzing methods

Participation in a scanner experiment can induce not only anxiety [1, 2, 3,4], but could also be accompanied by a hemispheric asymmetry of BOLD signals [5]. In order to explain such asymmetry, it is necessary to analyze state anxiety within the scanner in different resting states and thus to evaluate individual differences in anxiety processing. A physiological correlate of anxiety is HRV, whereby an increased HRV potentially constitutes an important resource for successful processing of unpleasant feelings, as for example anxiety [18]. Specific frequency components reflect different sources of HRV, such as the baroreceptor mediated blood pressure regulation and central commands. Hence, analyzing HRV frequency components not only allows to monitor individual changes of anxiety, but also to uncover details about their origin.

## Results

### State anxiety and anxiety change

**Fig 1:**
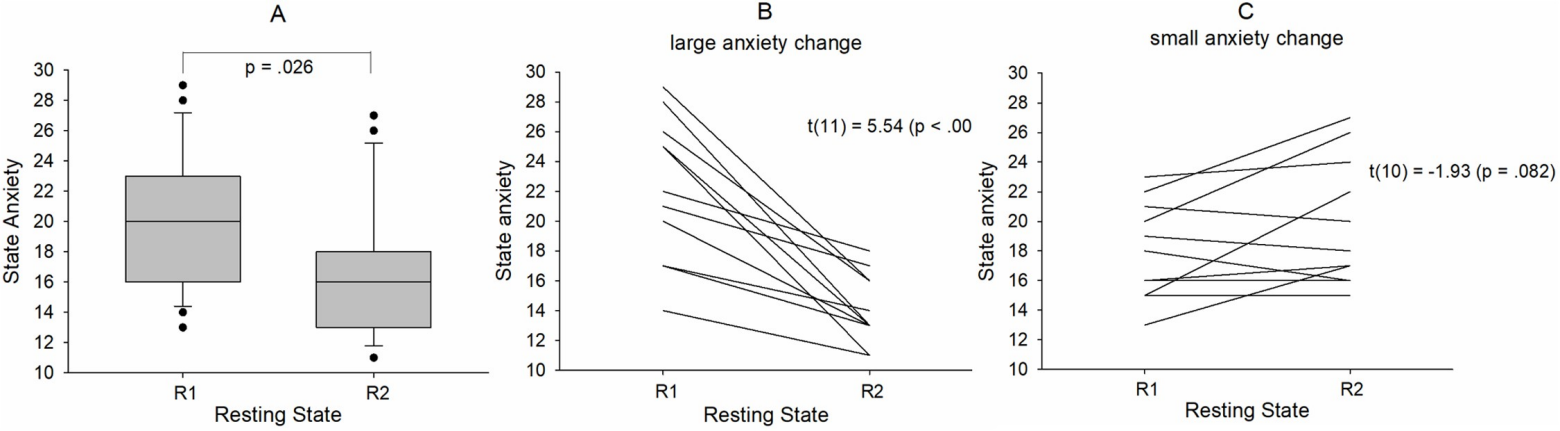
Boxplot depicting the distribution of state anxiety across the resting states R1 and R2 for all 23 subjects (A). Note: whiskers indicate the 10^th^ and 90^th^ percentile. Examples of strong anxiety decline (large anxiety change) in a group of 12 subjects (B) and weak anxiety decline or anxiety increase (small anxiety change) in a group of 11 subjects (C).

State anxiety varied across participants and resting states R1 and R2 in a broad range between 11 (low anxiety) and 29 (rather high anxiety) and significantly declined from R1 (AS1 = 19.96 ± 4.5) to R2 (AS2 = 16.83 ± 4.41) (t(22) = 2.39 (p = .026)) (Fig 1A). Of note, initial state anxiety (AS1) was significantly higher than in the normative sample [19] namely M = 19.96, SD = 4.5 vs. M_normative sample_ = 16.3, t(22) = 3.88, p = 0.001, thus suggesting comparably high levels of anxiety in the first resting state R1.

An interesting aspect of anxiety processing in consecutive resting states is the change of anxiety. The anxiety change (AC) is defined as difference of anxiety scores (possible range of AS scores: 10–40) in two resting states. For instance, with AS1 = 28 in R1 and AS2 = 13 in R2 AC equals -15. We found subjects with constant anxiety in R1 and R2 (AC = 0), anxiety decrease down to AC = -15 and anxiety increase up to AC = 7. To emphasize the difference between large and small (inclusive zero) anxiety change, two diagrams with the corresponding anxiety changes between R1 and R2 are displayed in Fig 1B (large AC) and Fig 1C (small AC).

### Relationship between BOLD signals, heart rate intervals (RRI) and respiration

**Fig 2:**
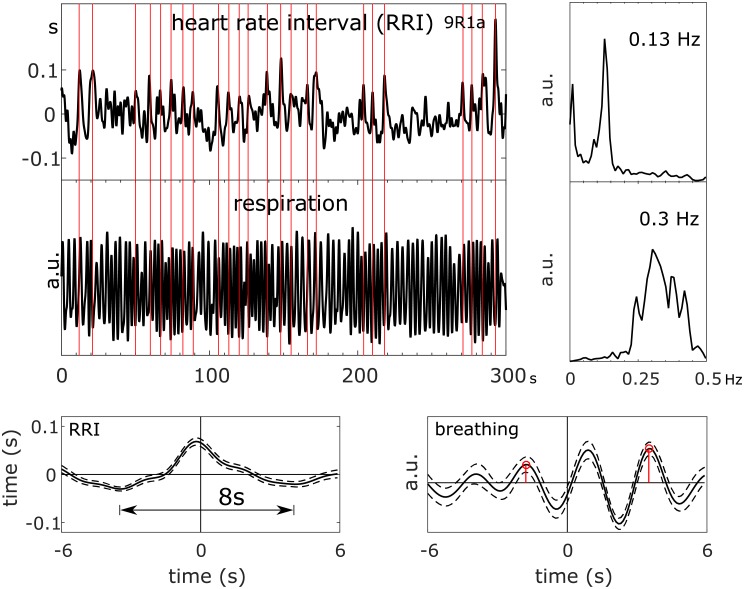
RRI time course, BOLD signals from PCG and insula and respiration (top left), corresponding power spectra (top right), and RRI peak-triggered averages (± SE; bottom) of RRI, BOLD and breathing signals from one subject (9R1; anxiety score AS1 = 23) with neural BOLD oscillations (pTD) in resting state R1 and high anxiety. The vertical lines indicate trigger and RRI peak maxima, respectively.

To give the reader an example of the data studied, trajectories of BOLD, breathing and RRI signals in one member of the small anxiety change group (9R1), raw signals, spectra and averaged waves are summarized in Fig 2. Because of the lack of triggers in resting state data, RRI peak-triggered averaging was used [7]. To obtain triggers, dominant peaks in the RRI time course are marked (indicated by vertical lines in Fig 2) and used as trigger to compute average waves of 12-s length (with 6 s before the trigger) for RRI, breathing and BOLD signals. The example depicts spectral peaks at 0.13 Hz in BOLD and RRI signals and a broad peak at 0.3 Hz in the spectrum of respiration. This broad spectral peak underlines the instability of the breathing signal with 2 or 3 breaths between two consecutive RRI peaks, and the averaged breathing wave refers to a phase coupling between slow RRI (0.13 Hz) and breathing (~ 0.3 Hz) oscillations. This example suggests the existence of neural BOLD signals (RRI precedes BOLD oscillations; [7]) with frequencies f > 0.1 Hz in resting states and documents the traveling of neural BOLD waves downwards (descending BOLD oscillations) from PCG (ROI 1) to insula (ROI 29) within ~ 1.5–0.9 = 0.6 seconds.

### Lateralization of phase coupling between BOLD oscillations at 0.1 Hz in precentral gyrus and insula

From each of the 23 individuals, two pairs of PLV parameters (delay and %sigbins), one from each hemisphere, were extracted for both resting states (R1, R2). The grand averages of the parameters delay and %sigbins (mean ± SD), separated for each hemisphere and resting state are summarized in Table 1. In addition, the significance (t-test) of hemispheric differences is indicated. Remarkably, the time delays revealed as significant lateralization with larger nTD values in the left hemisphere most pronounced in R1. The mean duration of significant phase coupling (%sigbins) between BOLD oscillations varied between 38%–53% and was also larger on the left side. Noteworthy, the most significant hemispheric asymmetry in R1 was associated with the highest state anxiety in R1.

**Table 1:** Mean (M), standard deviation (SD) and inter-hemispheric difference (Diff.) of time delay and %sigbins in 23 subjects.

		Left hemisphere		Right hemisphere		*Diff*.	*t*	*df*	*p*
		*M*	*SD*	*M*	*SD*				
R1	time delay (s)	-.39	.48	-.06	.62	-.33	-3.26	22	.004
R1	%sigbins (%)	52.5	25.7	38.0	22.8	14.5	3.09	22	.005
R2	time delay (s)	-.20	.54	.08	.64	-.28	-2.16	22	.04
R2	%sigbins (%)	45.6	19.6	38.3	23.8	7.30	1.86	22	.08

### Relationship between state anxiety, anxiety change and phase coupling of slow BOLD oscillations

For both hemispheres the Spearman correlations between state anxiety (R1, R2), anxiety change and significant phase coupling (positive and negative TD) of BOLD oscillations in precentral gyrus and insula were calculated (Table 2). In addition to the correlations between TD and anxiety for the whole sample (n = 23), the correlation for subjects with negative TD only (nTD; n = 18) was also calculated. Significant correlations were found between TD (R1) and anxiety change for the left hemisphere (r = 0.65, p = 0.001), nTD (R1) and anxiety change for left hemisphere (r = 0.53, p = 0.03), and TD versus state anxiety for the left hemisphere in R2 (r = 0.47, p = 0.03). The correlations between TD and state anxiety for the left and right hemispheres in R1 were marginally significant (r = -0.38, p = 0.074; r = -0.39, p = 0.069). With respect to the correlations between TD and state anxiety (R1, R2) it is remarkable that the signs of the correlations are different for both resting states, suggesting that both nTD in R1and pTD in R2 could play a prominent role for the processing of anxiety. Additionally, a Spearman correlation was calculated between framewise displacement (FD) and anxiety change. No significant correlation was found (r = -0.31, p = 0.15).

**Table 2:** Spearman rho correlations of time delays (TD; precentral gyrus and insula) for both hemispheres and negative time delays (nTD) for left hemisphere with both state anxiety (R1, R2, respectively) and anxiety change.

	n	State anxiety		Anxiety change
		R1	R2	
Left hemisphere				
TD	23	-.38 (p = .07)	.47 (p = .03)	.65 (p = .001)
nTD	18	-.39 (p = .11)	.28 (p = .26)	.53 (p = .03)
Right hemisphere				
TD	23	-.39 (p = .07)	.05 (p = .83)	.35 (p = .10)

**Fig 3:**
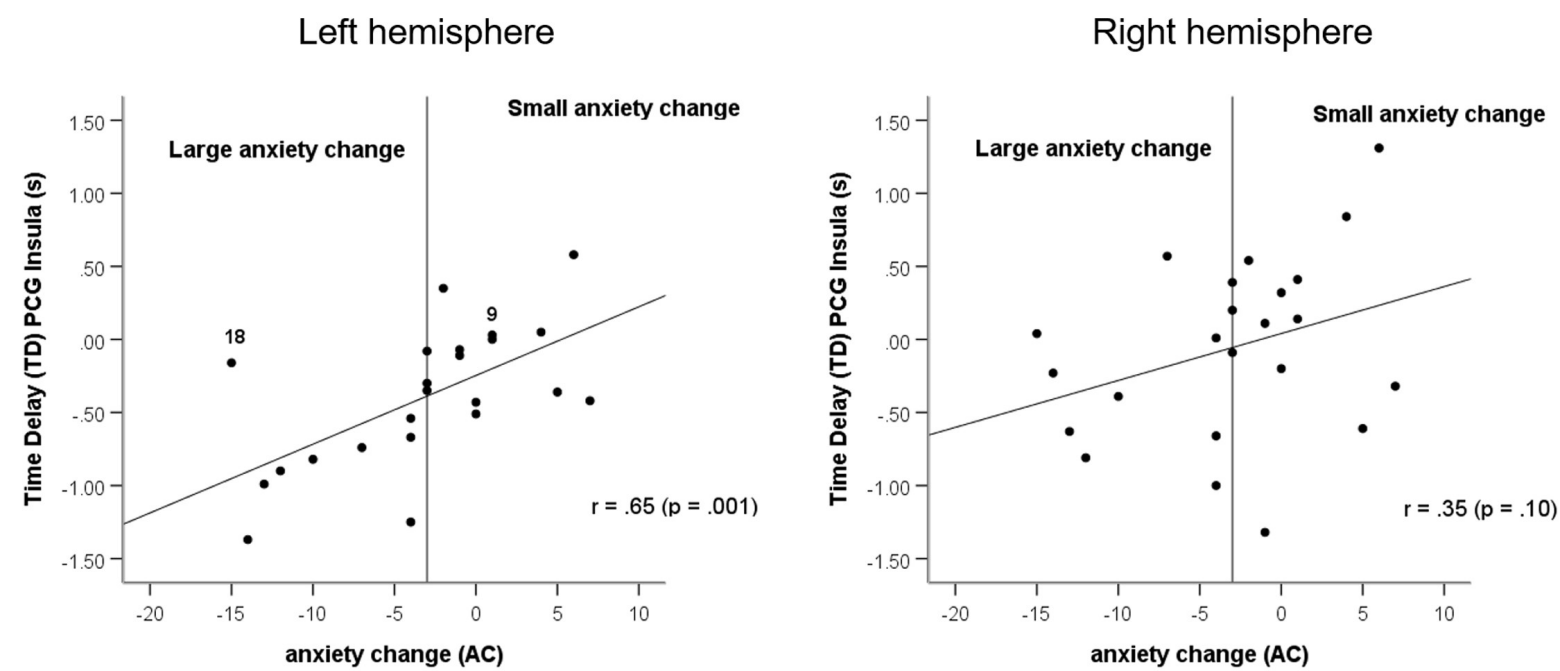
Relationship between anxiety change (AC) and significant negative or positive time delay (pTD, nTD) of BOLD oscillations in left precentral gyrus and left insula (left side) and right precentral gyrus and right insula (right side). TD data from resting state R1. The arbitrary threshold is indicated by a vertical line. Two subjects (9R1 and 18R1; left side) are indicated.

The diagram with significant correlation between anxiety change and phase coupling of BOLD oscillations (Fig 3, left side) indicates that different types of anxiety processing can be discriminated. The application of a cluster analysis revealed no clear threshold for the discrimination between two groups, because of their non-homogeneity. Thus, we introduced an arbitrary threshold of AC = -3. The group *large anxiety change* (AC< = -3) contains subjects of high anxiety in R1 and low anxiety in R2 concentrated in the left lower part of Fig 3, left side and the group *small anxiety change* (AC>-3) includes subjects displaying nearly constant or even increasing anxiety (Fig 3, left side). It is important to note, that a nearly comparable anxiety change was found in subjects with low but also with high anxiety state in R1. The main difference between both subgroups is that the large change group is associated with only vascular BOLD oscillations (nTD) in the left hemisphere, while the small change group is associated with a mix of both, vascular (nTD) and neural BOLD oscillations (pTD).

### Relationship between anxiety change and HRV in low frequency bands below and above 0.1 Hz

**Fig 4:**
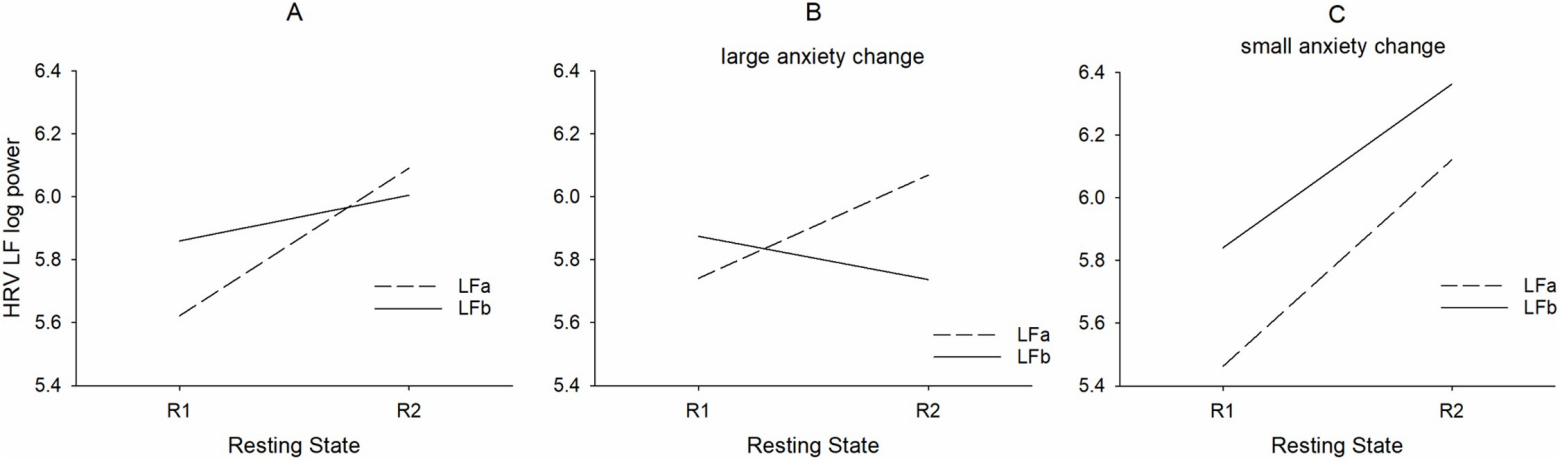
Interactions between resting states (R1, R2), LFa log power (stippled lines) and LFb log power (full lines) for all subjects (A), twelve subjects with *large change* (B) and nine subjects with *small change* (C).

The RRI data of one subject were missing in R2 and were disturbed in R1 in another subject, both belonging to the small change group. Therefore, only 9 subjects were left for analyses. The results for low frequency (LF) HRV changes from R1 to R2 are summarized in Fig 4A for all subjects, for subjects with *large anxiety change* (AC< = -3) in Fig 4B and for subjects with *small anxiety change* (AC >-3) in Fig 4C separately for LFa (0.06–0.1 Hz) and LFb (0.1–0.14 Hz). Due to multiple comparisons Bonferroni alpha adjustment was applied by dividing p = 0.05 by 4 (LFa and LFb for small anxiety change and large anxiety change groups, respectively), resulting in a threshold of p < 0.0125. The interaction between resting state x component (F(1,19) = 8.56, p = 0.009) was significant. This result indicates that both LFa power and LFb power increased from R1 to R2 (Fig 4A), while anxiety significantly decreased (Fig 1A). No significant interaction was found between component (LFa, LFb) x resting state (R1, R2) x group. Interestingly, there were between-group (large and small AC) differences in LFa and LFb power from R1 to R2. Whilst LFa power increased significantly (t(11) = 2.41, p = 0.035) in the large anxiety change group, both components, LFa power (t(8) = 2.69, p = 0.027) and LFb power (t(8) = 3.16, p = 0.013) increased significantly in the small change group.

### Relationships between neural BOLD oscillations and HRV in two narrow frequency bands below and above 0.1 Hz

**Fig 5:**
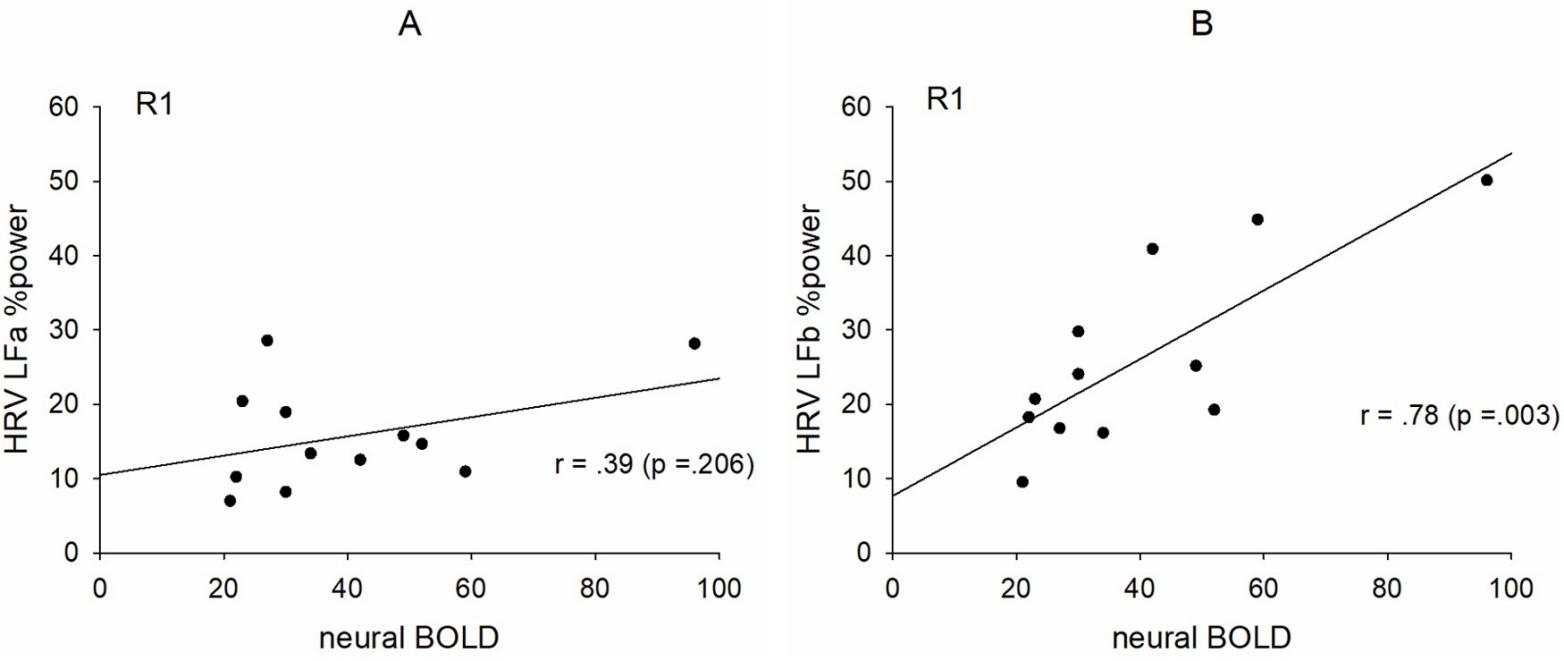
Relationships between HRV LF relative (%) power and percentage of significant phase coupling (%sigbins) between PCG and insula in the right hemisphere for neural BOLD oscillations (pTD). Low frequency %power in the band 0.06–0.1 Hz (LFa%) (A) and low frequency %power in the band 0.1–0.14 Hz (LFb%) (B).

In contrast to the left hemisphere, no correlation between anxiety change and phase coupling of slow BOLD oscillations was found for the right side, because of the non-dominance of vascular BOLD oscillations and the larger number of subjects with neural BOLD oscillations, respectively. If neural BOLD oscillations are associated with slow neural activity fluctuations, then a positive correlation between neural BOLD oscillations (%sigbins) and HRV LF %power is expected. The twelve subjects with pTD in R1 are indicated in the plots in Fig 5. Such a significant correlation was found, however, only for the LFb band (Fig 5) thus suggesting the existence of central commands preferably above 0.1 Hz.

## Discussion

The aim of this study was to explain the hemispheric asymmetry of intrinsic 0.1-Hz BOLD oscillations in healthy individuals without any former MRI experience by considering not only state anxiety and anxiety change between two consecutive resting states, but also the variation in time of the low frequency HRV in two bands. The discrimination between neural and vascular BOLD oscillations based on PLV calculation depends on several factors. The PLV can either be calculated between BOLD signals and HR interval (RRI) time courses [6,7] or between two BOLD signals [5], whereby the ROI selected for BOLD signal recording is a crucial issue. The latter method is of interest, because no ECG recording is necessary within the scanner and by choosing ROIs in the supply territory of a specific main cerebral artery, the vascular BOLD can be better defined. We monitored the BOLD signal in the supply territories of the left and right middle cerebral artery (MCA), in PCG and insula, two regions of great importance for conscious experience of intending to act [27] and control of cardiac function [28, 29]. The MCA is commonly used to measure cerebral blood flow velocity (CBFv) during rest and complex cognitive tasks as it supplies 80% of each respective cerebral hemisphere’s frontal lobe [30].

### MRI-related anxiety and anxiety change

It is known that anxiety is increased during the first scan and drops to normal levels later [3]. Surprisingly, the anxiety changes between two resting states separated by ~ 30 min shows considerably variation as documented in Fig 1B and 1C, either in form of a sharp anxiety decline (large change) or even a moderate anxiety increase (small change). These different trajectories in state anxiety are particularly interesting and suggest that different person-specific mechanisms of anxiety regulation might have been activated.

### Relationship between state anxiety, anxiety change and phase coupling of slow BOLD oscillations in the territory of the left MCA

Spontaneous BOLD oscillations around 0.1 Hz can be generated in parts by a complex interplay of slow cerebral blood volume and CBF oscillations [31] and by intrinsic neural activity fluctuations [15]. The former is closely associated to the CBFv measured by transcranial Doppler sonography in the main cerebral arteries and phase-coupled with blood pressure waves measured via infrared finger plethysmography [13, 32]. In healthy subjects and rest, the CBFv of both MCAs oscillates nearly synchronously and demonstrates hemispheric symmetry [30, 32]. Intrinsic neural activity fluctuation around 0.1 Hz can be observed in EEG or ECoG recordings and have been reported in the beta/alpha power at EEG electrodes over sensorimotor areas [33] and in the beta and gamma power at multiple electrode sites placed in human posteromedial cortex [34]. The close relationship between gamma power and BOLD oscillations (neural BOLD) was documented impressively by Mateo et al. [15] via entrainment of arteriole vasomotor fluctuations by neural activity.

Both types of slow BOLD oscillations have a different origin and can thus be composed by different frequency components. Our assumption was that a discrimination should be possible by their direction of spreading, vascular BOLD oscillations are ascending in the supply territory of MCA and neural BOLD oscillations are descending from higher centers in direction to the cardiovascular centers in the brain stem ultimately modulating the heart rate [18]. The trend toward larger nTD magnitudes with a stronger decline of anxiety (Fig 3, left side) may be interpreted as accumulating dominance of vascular BOLD oscillations. The opposite, the trend toward larger pTD magnitudes may be indicative for an accumulating dominance of neural BOLD oscillations. Both, the significant correlation between anxiety change and phase coupling (pTD, nTD) in the left hemisphere (Fig 3, left side) and the significant hemispheric asymmetry with the predominance of nTD in the left side (Table 1) provide strong hints toward a cerebral blood flow increase in the territory of the left MCA, which could constitute a precondition for a successful processing of high anxiety states.

An interesting observation is the different sign of correlation between TD and state anxiety indicative for different trends in R1 and R2 in the left hemisphere (Table 2). A negative correlation underlines the dominance of vascular BOLD oscillations (nTD) in the first resting state R1, while the positive correlation stress the importance of neural BOLD oscillations (pTD) in R2. This also confirms the importance of left-sided blood flow increase in anxiety processing preferable in the first resting state.

Anxiety and/or fear is not only accompanied by activation of the left amygdala [8, 35] but also of the left insular cortex [4]. Dennis et al. [4] reported a 6-min resting state study on young and adult participants with scanning followed by questionnaires to assess their mood and thoughts during the scan. Both groups showed increased connectivity between left insular cortex and the default mode network [36] with increased anxiety. This left-sided insular activation during task-free periods suggests that increased state anxiety is reflected in resting-state functional connectivity. Positron emission tomography (PET) revealed an increase of cerebral blood flow during emotion induction in left orbitofrontal and left anterior insular cortices, which was correlated with HF-HRV [37]. Common in these reports [8,35,36] is the involvement of structures in the left hemisphere preferentially involved in regulating vagal tone. Together these findings suggest an increase in cerebral metabolic activity—but also its by-product carbon dioxide—in left amygdala, left insula and related structures especially during negative emotion processing. This in turn leads to a local increase of CBF to supply the areas with oxygenated blood and remove waste products [38]. The lateralized increase of cerebral blood flow circulation in the left MCA in the majority of subjects with large anxiety change is therefore not unexpected and contributes to a concomitant increase in low frequency HRV.

### Relationship between anxiety change and HRV in low frequency bands below and above 0.1 Hz

An interesting finding refers to the relationship between state anxiety and HRV. A combined BOLD-HRV study in patients with posttraumatic stress disorders and healthy controls revealed higher low frequency (LF) HRV and increased connectivity between left amygdala and periaqueductal gray in controls [39]. In this case the standard LF band (0.04–0.15 Hz; Task Force Guidelines 1996) was used. Because of the LF-HRV difference between patients and controls with higher anxiety in the former, an increase of LF-HRV could be expected from R1 to R2 in healthy scanner-naive subjects.

Studies using HRV as a biomarker for stress, pain, anxiety and other unpleasant emotions ([17, 18, 37, 40, 41]) report on two major components, the HF component (0.15–0.4 Hz) being sensitive to vagal efference and the LF component (0.01–0.15 Hz or 0.04–0.15 Hz) reflecting both sympathetic and parasympathetic efferences. To our knowledge there is no report differentiating between different LF components, although the LF component depends on a mixture of both parasympathetic and sympathetic autonomic influences. The existence of two distinguishable low frequency bands is confirmed by a spectral analysis of HR and BP signals [42] suggesting two principal frequency components at 0.08 Hz and 0.12 Hz. However, their underlying generating mechanisms were not elucidated. The discrimination between HRV frequency bands below (LFa: 0.06–0.1 Hz) and above 0.1 Hz (LFb: 0.1–0.14 Hz) is of multiple interest and leads among others to the following questions: Is any of these frequency bands predominant in the context of the *pacemaker theory* proposed by Julien [43] assuming the existence of rhythmic central commands? Can the discrimination between two LF HRV components help to differentiate between diverging anxiety processing strategies? Is one band more indicative for a parasympathetic influence?

Considering the different and variable patterns associated with a large anxiety decrease in one group and no change or even an anxiety increase in the other group (examples see Fig 1B and 1C), it is not astonishing that no significant interaction was found between resting state, group and component. The significant interaction between resting state and component documents the expected increase of LF HRV in connection with a general decline in anxiety from R1 to R2 (Fig 4A). Noteworthy, there was also a significant interaction between resting state and group (Fig 4A and 4C) with a significant increase of both LF components from R1 to R2 in the small change group and an increase of only LFa power in the large change group. It might thus be concluded that frequency components between 0.1–0.14 Hz may play a minor role in subjects with large anxiety change, however a major role in subjects with stable anxiety scores. Considering both the HRV analysis and the correlation between anxiety change and BOLD phase-coupling, it might be speculated that one mechanism of anxiety processing is based on enhanced vascular BOLD oscillations (*baroreflex theory*) predominant in the band 0.06–0.1 Hz, and the other mechanism is based on reinforced rhythmic central commands (*pacemaker theory*) with a frequency preference in the 0.1–0.14 Hz band. These central commands originating in prefrontal cortex control cardiac activity via vagus nerve activation [29]. Herewith the parasympathetic influence in the 0.1–0.14 Hz band may be explained by a vagally-mediated RRI increase (HR deceleration). Further research is necessary however, to verify or falsify this hypothesis.

### Relationships between neural BOLD oscillations and low frequency band HRV

The majority of neural BOLD oscillations (pTD) was found in the right hemisphere in R1. Such descending neural BOLD oscillations were observed in twelve subjects. If these oscillations are associated with central commands projecting to the cardiovascular nuclei in the brain stem and are responsible for the fast modulation of HR, then there should emerge a correlation between the length of significant phase coupling (%sigbins) in descending BOLD oscillations and LF HRV. The results in Fig 5 indicate that such a correlation is likely, however, only for the LFb band (0.1–0.14 Hz). This finding not only confirms the reported phase-coupling between neural BOLD oscillations and RRI signals [7], but provides evidence that neural BOLD oscillations are not only restricted to frequency components below 0.1 Hz [44], thus suggesting that central commands operate dominantly at frequencies above 0.1 Hz.

### Conclusions

1. Healthy, scanner-naïve individuals displayed MRI-related state anxiety ether with a strong anxiety decline (large anxiety change) between two resting states at intervals of ~30 minutes or a moderate anxiety change (small change). This suggests that there were different mechanism of anxiety processing within the sample.
2. Measuring the phase-coupling between intrinsic BOLD oscillations at 0.1 Hz in precentral gyrus and insula allows to discriminate between vascular and neural BOLD oscillations and moreover, to differentiate between subjects with different trajectories of state anxiety. Subjects with a large anxiety change revealed a hemispheric asymmetry especially during the first resting state with a predominance of vascular BOLD oscillations at 0.1 Hz in the left hemisphere and subjects with rather stable anxiety showed a dominance of intrinsic neural BOLD oscillations at 0.1 Hz.
3. The analysis of within-scanner HRV revealed a pronounced increase of low frequency power between both resting states. This power increase was dominant in the band 0.06–0.1 Hz in the large change group and in the band 0.1–0.14 Hz in the small change group and exhibits that rhythmic central commands modulating the heart rate via vagus nerve activation operate dominantly at frequencies above 0.1 Hz.

### Limitations

Some limitations of this research need to be emphasized. First, the small sample size limits the robustness of the findings and calls for replication studies. Second, the number of non-reliable pTD or nTD values (e.g. %sigbins < 10%) increased with proceeded scanning time, thus the use of only significant PLV measures (p<0.05) may have been too conservative. Third, the use of only two pairs of BOLD oscillations in the territory of MCA and two resting states limits the interpretation of the findings. To overcome these problems it is recommended to use a large number of ROI pairs in prefrontal and parietal cortices including the limbic system with the amygdala, all four resting states and instead of only significant PLV values all PLV values (this work is in progress).

Fourth, it should be noted that patterns of PLV (nTD or pTD) are not subject-specific and not stable across resting states. They can change from nTD in resting state R1 to pTD in R2 and vice versa. This unstable behavior has already been reported earlier and denoted as “switching phenomenon” [6]. It could be speculated that in cases of a high anxiety level not only one, but both mechanisms (blood flow increase and central commands) become activated. An example for such a pattern is subject 18R1 classified as member of the large drop group and marked as outlier in Fig 3 (left side). This subject displayed two peaks in the HRV spectrum one at 0.09 Hz and the other at 0.11 Hz. The dominance of HRV power in the 0.1–0.14 Hz band in the initial resting state R1 with high anxiety score (AS = 28) suggests that in addition to the blood flow increase also strong central commands were active.

Finally, the high inter-and intra-subject variability of breathing patterns and the selection of LF and HF HRV bands could be of concern. Breathing is regulated either by respiratory neurons in medulla oblongata and pons (metabolic breathing) or from higher centers in the cerebral cortex (behavioral breathing) and can be conscious (thinking about the breath) or unconscious [45, 46]. In our study no instruction was given about the type of respiration. The majority of participants displayed spontaneous breathing rates of 0.2–0.4 Hz, a minority displayed breathing at 0.1 Hz (6/min) especially in the first resting state R1 where state anxiety was highest. While breathing at a rate between 0.2–0.4 Hz contributes to the HF-HRV band, breathing at ~ 6/min facilitates the LF band. One solution of the problem could be a controlled breathing at a subject-specific rate in the HF band.
