# Correction: MRI-related anxiety in healthy individuals, intrinsic BOLD oscillations at 0.1 Hz in precentral gyrus and insula, and heart rate variability in low frequency bands

**DOI:** 10.1371/journal.pone.0216731

**Published:** 2019-05-07

**Authors:** Gert Pfurtscheller, Andreas Schwerdtfeger, David Fink, Clemens Brunner, Christoph Stefan Aigner, Joana Brito, Alexandre Andrade

Fig 2, “RRI time course, BOLD signals from PCG and insula and respiration (top left), corresponding power spectra (top right), and RRI peak-triggered averages (± SE; bottom) of RRI, BOLD and breathing signals from one subject (9R1; anxiety score AS1 = 23) with neural BOLD oscillations (pTD) in resting state R1 and high anxiety,” is incorrect. Please see the complete, correct Fig 2 below.

**Fig 2:**
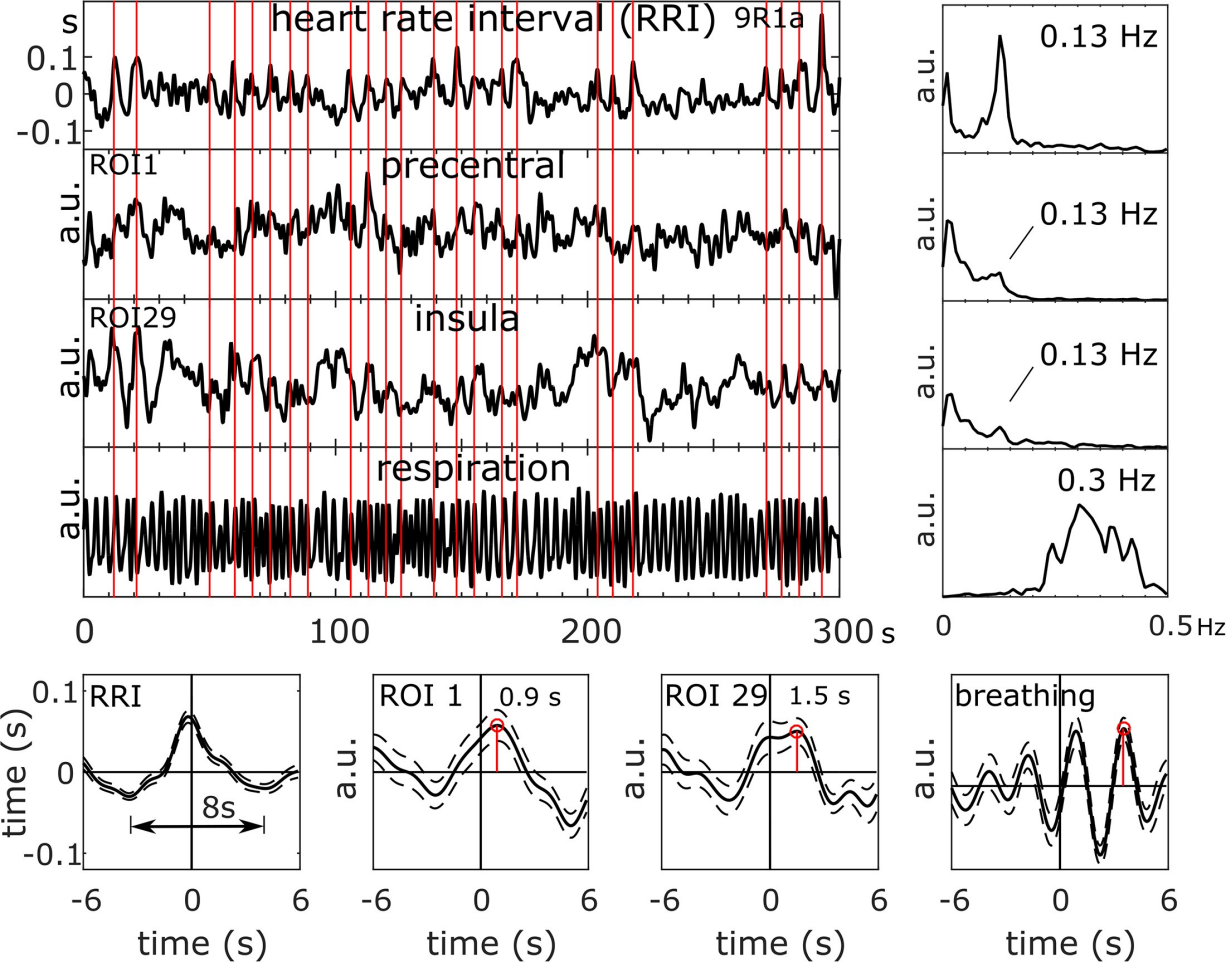
RRI time course, BOLD signals from PCG and insula and respiration (top left), corresponding power spectra (top right), and RRI peak-triggered averages (± SE; bottom) of RRI, BOLD and breathing signals from one subject (9R1; anxiety score AS1 = 23) with neural BOLD oscillations (pTD) in resting state R1 and high anxiety. The vertical lines indicate trigger and RRI peak maxima, respectively.
